# PD174 The Emerging Role Of National Health Service England In The UK – An Access Enabler Or Barrier For Innovations?

**DOI:** 10.1017/S0266462324004008

**Published:** 2025-01-07

**Authors:** Aigun Gassanova, Katherine Leong, Richard Macaulay

## Abstract

**Introduction:**

Since April 2017, National Health Service England (NHSE) has been granted new powers to negotiate directly with pharmaceutical companies offering innovative, high value medicines. This research systematically evaluated all innovative therapies that have undergone commercial discussions with NHSE.

**Methods:**

NHSE press releases for the period from 1 April 2017 to 10 November 2023 were screened for drug reimbursement decisions and the corresponding National Institute for Health and Care Excellence (NICE) assessments were identified. Key information was extracted, including reimbursement decision, date of decision, and type of commercial deal.

**Results:**

NHSE announced the conclusion of commercial discussions for 36 therapies (four in 2017, three in 2018, nine in 2019, two in 2020, nine in 2021, seven in 2022, and two in 2023). Of these, 27 NHSE commercial discussions were associated with positive NICE guidance; 12 of the 27 preceded the NICE final appraisal determination. In addition, three of the 36 therapies were associated with negative NICE appraisals (not recommended or non-submissions), one had an ongoing NICE assessment, and five did not go through NICE (subject to clinical commissioning policies). The specific type of agreement was not typically stated, but two were outcomes-based agreements, three were budget neutral, one was a portfolio-wide agreement, and one was a population health agreement.

**Conclusions:**

NHSE is becoming an increasingly active and important stakeholder in medicines access. The UK may become an increasingly important early launch market for certain therapies, as evidence by the Medicines and Healthcare products Regulatory Agency joining Project Orbis and the Access Consortium and Casgevy (the first CRISPR-based gene therapy) receiving its first global marketing authorization in the UK.

